# Association between body mass index and activities of daily living in homecare patients

**DOI:** 10.12669/pjms.336.13748

**Published:** 2017

**Authors:** Guzin Zeren Ozturk, Memet Taskın Egici, Mulazim Hussain Bukhari, Dilek Toprak

**Affiliations:** 1 Guzin Zeren Ozturk, Family Medicine Specialist, Sisli Hamidiye Etfal Training and Research Hospital, Family Medicine Clinic, Istanbul, Turkey; 2 Memet Taskın Egici, Family Medicine Specialist, Sisli Hamidiye Etfal Training and Research Hospital, Family Medicine Clinic, Istanbul, Turkey; 3 Mulazim Hussain Bukhari, HOD, Department of Pathology, University of Lahore, Lahore - Pakistan; 4 Dilek Toprak, Associate Professor, Family Medicine, Sisli Hamidiye Etfal Training and Research Hospital, Family Medicine Clinic, Istanbul, Turkey

**Keywords:** Aged, Body Mass Index, Homecare Patient, Homecare Services, Obesity, Quality of Life

## Abstract

**Objective:**

Overweight or obesity may cause many chronic illnesses. Furthermore, several studies have shown that high body mass index is associated with mortality and morbidity among the elderly. Therefore, obesity or being overweight could adversely affect the performance of activities of daily living. In this study our aim was to investigate the association between Body Mass Index and Activity of Daily Living in Homecare Patients.

**Method:**

The records of 2016 from the homecare unit of Sisli Hamidiye Etfal Training and Research Hospital were retrospectively reviewed. During this period, 1105 patients visited this facility. Unconscious or bedridden patients (hemiplegia, hemiparesia, and tetraparesis) and patients with incomplete data were excluded from the study. Therefore, the survey was completed with 250 files, which included all the data needed for our research. Age, gender, Body Mass Index and Barthel Index scores were recorded to the statistical program; p≤0.05 was considered as statistically significant.

**Results:**

One hundred fifty one (60.4%) were women, and 99 (39.6%) were men. The relations between gender and age, weight, and Barthel index scores were not statistically significant. There was a significant positive correlation between weight and Barthel index scores as well as between Body Mass Index and Barthel index scores (r = 0.190; p = 0.003). The patients were divided into two groups: Group-I (underweight and normal weight) and Group-II (overweight and obese). Group-II exhibited a much higher ability to perform Activity of Daily Living than Group-I (p = 0.002).

**Conclusion:**

Some studies report that obesity is protective against Activity of Daily Living, but the opposite is reported in some others. Our study showed increased values of Body Mass Index and Activity of Daily Living ability, which are indicative of protective effects. The relationship between Body Mass Index and physical disability is not yet proven to be linear.

## INTRODUCTION

Overweight or obesity may cause many chronic illnesses. Furthermore, several studies have shown that high body mass index (BMI) is associated with mortality and morbidity among the elderly.^1^ Mobility is another important topic in relation to the elderly. Difficulties in mobility are often the first sign of functional decline and may indicate the need for preventive measures.^2^ Mobility problems have been reported as a predictor of all-cause mortality, and patients with BMI >30 kg/m^2^ have low scores in the “Time Up to Go” test, which assesses mobility.^3^ Mobility problems as well as illnesses leading to cognitive impairment are a cause of dependence. Weight loss through diet may be associated with cognitive improvement in patients with mild cognitive impairment.^4^ Therefore, obesity or being overweight could adversely affect the performance of activities of daily living (ADLs).

Barthel index is a simple index of independence, which is used to score the patients’ ability to perform ADLs. Since 1955, this index has been used in hospitals in Maryland for patients with chronic diseases.^5^ The Barthel index comprises 10 items, including the presence or absence of fecal and urinary incontinence and the need for assistance with grooming, toilet use, feeding, transfers (e.g., from chair to bed), walking, dressing, climbing stairs, and bathing. In this study, we investigated the association between BMI and ADLs in homecare patients.

## METHODS

The records for the period between 01 January 2016 and 31 December 2016 from the homecare unit of Sisli Hamidiye Etfal Training and Research Hospital were retrospectively reviewed. During this period, 1105 patients visited this facility. Unconscious or bedridden patients (hemiplegia, hemiparesia, and tetraparesis) and patients with incomplete data were excluded from the study. Therefore, the survey was completed with 250 files, which included all the data needed for our research. BMI is defined as the body mass divided by the square of the body height, is universally expressed in units of kg/m^2^, and is classified as follows.

- Underweight<18.50 kg/m^2^
- Normal range18.50 ≤ X ≤ 24.99 kg/m^2^
- Overweight≥25.00 kg/m^2^
- Obese ≥30.00 kg/m^2^

The Barthel index was used as a screening tool to assess the ability to perform ADLs. The Barthel index scores are in multiples of five, ranging from 0 (completely dependent) to 100 (independent in basic). Higher scores represent a higher degree of independence.

The Barthel index scores are classified as follows:

- 0–20 points: total dependency
- 21–60 points: high-level dependency
- 61–90 points: mid-level dependency
- 91–99 points: low-level dependency
- 100 points: total independence

Age, gender, chronic diseases, BMI, and Barthel index scores were analyzed using a statistical program. According to the Shapiro–Wilk test, our study population had an abnormal distribution (p < 0.001). The Mann–Whitney U test was used to compare independent variables between groups. Chi-square was used to analyze relations between two non-continuous variables; for continuous variables, the correlation test was used. P ≤ 0.05 was considered statistically significant.

## RESULTS

**Fig.1:**
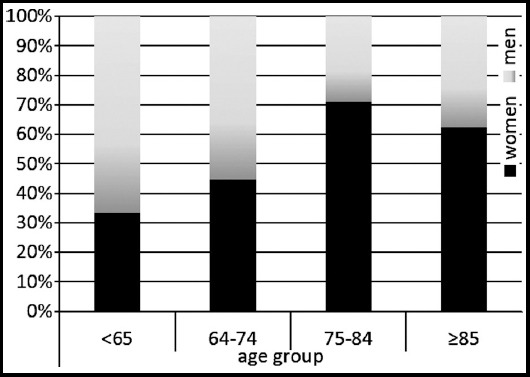
Distribution of age groups according to gender.

**Fig.2:**
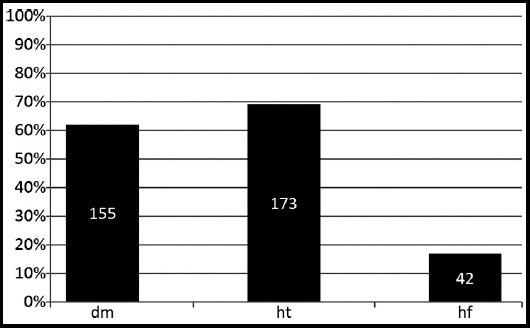
Distribution of chronic diseases

Two hundred fifty patients were included in our study: 151(60.4%) were women and 99 (39.6%) were men. The patient age groups and gender distributions are shown in Fig.1. The number of women aged >65 years was much greater than that of men, and hypertension was the most commonly occurring chronic disease (n = 173; 60%; Fig.2).

**Fig.3:**
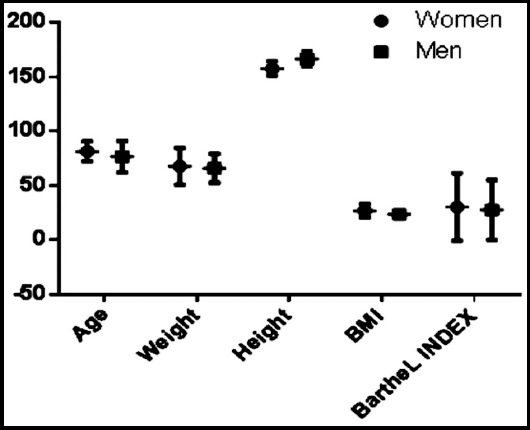
Age, weight, height, BMI and Barthel index scores stratified by gender

Age, weight, height, BMI, and Barthel index scores stratified by gender are shown in Fig.3. The relations between gender and age, weight, and Barthel index scores were not statistically significant (p = 0.050, 0.538, and 0.587, respectively). However, the men were taller than the women (p = 0.000), and the women had higher BMIs than the men (p = 0.000). Gender-based comparisons revealed significant correlations between BMI and Barthel index scores only among women (r = 0.299; p = 0.00).

There were significant negative correlations between age and weight, height, Barthel index scores (i.e., when age increased, the weight, height, and Barthel index scores decreased). Table-I There was a significant positive correlation between weight and Barthel index scores as well as between BMI and Barthel index scores (r = 0.190; p = 0.003). Therefore, when age increased, weight, BMI, Barthel index scores, and ability to perform ADLs decreased.

**Table-I:** Correlations among age, weight, height, BMI, and Barthel index scores.

*Correlations*						
Age	Pearson Correlation	1	-	-	-	-
	Sig. (2-tailed)					
Weight	Pearson Correlation	-0.183**	1	-	-	-
	Sig. (2-tailed)	0.004				
Height	Pearson Correlation	-0.207**	0.416**	-	-	-
	Sig. (2-tailed)	0.001	0.000			
BMI	Pearson Correlation	-0.111	0.898**	0.032	1	-
	Sig. (2-tailed)	0.080	0.000	0.612		
Barthel Index	Pearson Correlation	-0.149*	0.163*	0.037	0.190**	1
	Sig. (2-tailed)	0.019	.010	0.565	0.003
		Age	Weight	Height	BMI	Barthel Index

** . Correlation is significant at the 0.01 level (2-tailed).

* . Correlation is significant at the 0.05 level (2-tailed).

All the items of the Barthel index were not correlated with BMI. Assistance with feeding, toilet use, dressing, climbing stairs, bathing, and walking as well as urinary and fecal continence were positively correlated with BMI. As shown in Table-II, need for assistance with grooming and need for transfers (e.g., from chair to bed) using a wheelchair were not correlated.

**Table-II:** Correlations between items of the Barthel index and BMIs.

NT	Sig. (2-tailed)	-	-	-	-	-	-	-	-	-	-	-	-
	Pearson Correlation	0.780**	1	-	-	-	-	-	-	-	-	-	-
	Sig. (2-tailed)	0.000	-	-	-	-	-	-	-	-	-	-	-
G	Pearson Correlation	0.713**	0.641**	1	-	-	-	-	-	-	-	-	-
	Sig. (2-tailed)	0.000	0.000		-	-	-	-	-	-	-	-	-
T	Pearson Correlation	0.661**	0.575**	0.627**	1	-	-	-	-	-	-	-	-
	Sig. (2-tailed)	0.000	0.000	0.000		-	-	-	-	-	-	-	-
B	Pearson Correlation	0.385**	0.410**	0.363**	0.608**	1	-	-	-	-	-	-	-
	Sig. (2-tailed)	0.000	0.000	0.000	0.000		-	-	-	-	-	-	-
W	Pearson Correlation	0.450**	0.286**	0.542**	0.577**	0.473**	1	-	-	-	-	-	-
	Sig. (2-tailed)	0.000	0.000	0.000	0.000	0.000		-	-	-	-	-	-
WC	Pearson Correlation	0.146*	0.093	−0.096	0.296**	0.014	−0.151*	1	-	-	-	-	-
	Sig. (2-tailed)	0.021	0.142	0.129	0.000	0.828	0.017		-	-	-	-	-
CS	Pearson Correlation	0.429**	0.356**	0.379**	0.644**	0.517**	0.542**	0.194**	1	-	-	-	-
	Sig. (2-tailed)	0.000	0.000	0.000	0.000	0.000	0.000	0.002		-	-	-	-
D	Pearson Correlation	0.669**	0.560**	0.650**	0.836**	0.591**	0.623**	0.236**	0.701**	1	-	-	-
	Sig. (2-tailed)	0.000	0.000	0.000	0.000	0.000	0.000	0.000	0.000		-		-
UC	Pearson Correlation	0.802**	0.652**	0.653**	0.754**	0.570**	0.531**	0.214**	0.620**	0.805**	1	-	-
	Sig. (2-tailed)	0.000	0.000	0.000	0.000	0.000	0.000	0.001	0.000	0.000		-	-
FC	Pearson Correlation	0.791**	0.658**	0.628**	0.779**	0.585**	0.499**	0.253**	0.632**	0.788**	0.902**	1	-
	Sig. (2-tailed)	0.000	0.000	0.000	0.000	0.000	0.000	0.000	0.000	0.000	0.000		-
Bmı	Pearson Correlation	0.138*	0.009	0.168**	0.223**	0.106	0.251**	0.092	0.103	0.183**	0.143*	0.164**	1
	Sig. (2-tailed)	0.029	0.886	0.008	0.000	0.095	0.000	0.146	0.104	0.004	0.023	0.009	
	N	250	250	250	250	250	250	250	250	250	250	250	250
		Feeding	NT	G	T	B	W	WC	CS	D	UC	FC	BMI

** . Correlation is significant at the 0.01 level (2-tailed)

* . Correlation is significant at the 0.05 level (2-tailed).

Help needed with feeding (F), transfers (NT), grooming (G), toilet use (T), bathing (B), walking (W), wheelchair use (WC), climbing stairs (CS), and dressing (D) as well as fecal continence (FC) and urinary continence (UC).

According to their BMIs, the patients were classified as underweight (n = 10; 4%), normal weight (n = 125; 50%), overweight (n = 76; 30.4%), and obese (n = 39; 15.6%). The mean Barthel index scores stratified by BMI were as follows: underweight, 26.50 ± 20; normal weight, 22.93 ± 27.13; overweight, 31.51 ± 31.86, and obese, 42.30 ± 31.28. The patients were divided into two groups: Group-I (underweight and normal weight) and Group-II (overweight and obese). Group-II exhibited a much higher ability to perform ADLs than Group-I (p = 0.002).In addition, 28 (11.2%) of our patients died in 2016, 15 (53.6%) of whom were women. We identified no relation between death and gender (p = 0.433). Furthermore, there was no relation between death and age, BMI, and Barthel index scores (p = 0.482, 0.737, and 0.288, respectively).

## DISCUSSION

According to the World Health Organization, 71.4 years (males: 69.1 years; females: 73.7 years) is the average life expectancy at birth, and the life expectancy over the age of 60 years was 20.4 years (males: 18.9 years; females: 21.7 years) among the global population in 2015.^6^ In Turkey, the life expectancy at birth is 66.2 years.^6^ According to our national statistics, the elderly population was 6,651,503 in 2016 and accounted for 8.3% of all populations (males: 43.9%; females: 56.1%).^7^ Similarly, 60.4% of our study group was female, which is probably the result of the longer life expectancy of women.

According to the Turkey Statistical Institute, 61.5% of the elderly population are in the age group of 65–74 years, 30.2% are in the age group of 75–84 years, and 8.2% are in the age of ≥85 years.^8^ Several studies have shown that most homecare patients are aged >65 years.^9^,^10^ In our study, 16.6% of the elderly population was in the age group of 65–74 years, 39.3% were in the age group of 75–84 years, and 44.1% were in the age group of ≥85 years. These findings are because the need for homecare increases with age; accordingly, most of our study population was aged >85 years.

Chronic illnesses are long-term illnesses and usually incurable. The World Health Organization has listed cardiovascular accidents (stroke), cancer, chronic obstructive pulmonary disease, and diabetes as the most prevalent chronic illnesses worldwide.^11^ According to the cause of death statistics in Turkey, 46.3% of elderly people died from circulatory system diseases in 2015.^8^ In a study of a 3-year (2003–2006) Canadian homecare data, the most frequent chronic illness among 149,378 long-term homecare patients was chronic renal disorder, followed by hypertension, diabetes, heart failure, and depression.^12^ A study in Turkey revealed that hypertension was detected in 41.8% of homecare patients.^13^ In our study, hypertension was the most frequently observed chronic disease (n = 173; 60%). Moreover, hypertension is observed 30%–45% of the general population, and this percentage increases with age.^14^

The prevalence of overweight and obesity is increasing in the elderly population.^15^ Obesity is known to have a negative effect on mortality and morbidity.^16^,^17^ Numerous cohort studies have reported that higher levels of frailty are predicted by not only cognitive impairment but also various frailty-related indicators such as BMI and ADL-performing ability.^18^,^19^ However, we identified a positive correlation between BMI and ADL-performing ability in our population. Similarly, we obtained controversial results for whether overweight increases the ADL disability^20^ or has protective effects.^21^ This is because ADL-performing ability changes with not only weight but also eating habits, and the eating habits of overweight or obese individuals may decrease the malnutrition risk and may protect against the decline of ADL ability, given that nutritional status is related to ADL-performing ability in geriatric patients.^22^

Overweight and obesity, as measured by BMI, are also associated with a higher probability of ADL disability among women but not men.^23^ By contrast, our study showed that ADL-performing ability in women positively correlated with BMI. This is because elderly individuals lose approximately 1% of their lean mass (mainly muscle mass) per /year, with men losing more muscle mass than do women, both in absolute and relative terms.^24^ Hence, increased BMI led to increased ADL-performing ability among women in our study.

Many studies have shown that sarcopenia which is one of the main problems in the elderly, is associated with the decline in muscle mass and strength and is a predictor of poor outcomes, including mortality, disability, and poor quality of life.^24^ Malnutrition and weight loss are the causes of sarcopenia which is associated with functional dependence in the elderly.^25^ Accordingly, in our study, when the BMI decreased, the ADL-performing ability decreased, possibly because of the slowing down of activity due to malnutrition or sarcopenia.

## CONCLUSION

The relationship between obesity and ADL in the literature is unclear. Some studies report that obesity is protective against ADL, but the opposite is reported in some others. In our study showed increased values of BMI and ADL ability, which are indicative of protective effects. The relationship between BMI and physical disability is not yet proven to be linear. This is because underweight and obesity have similar risks. Accordingly, we conclude that overweight, obesity, and female gender are associated with an increase in ADL-performing ability in some cases. In general, physicians can advise all patients to receive adequate nutrition and perform exercises, which will protect them from most chronic diseases and help them with healthy aging.

### Author`s Contribution

**GZO:** Main author is responsible for the integrity of the study data.

**MTE:** Helped in collection of data.

**MHB & DT:** Helped in preparing the manuscript.
